# Quality of interaction between primary health-care providers and patients with type 2 diabetes in Muscat, Oman: an observational study

**DOI:** 10.1186/1471-2296-7-72

**Published:** 2006-12-07

**Authors:** Nadia Abdulhadi, Mohammed Ali Al-Shafaee, Claes-Göran Östenson, Åsa Vernby, Rolf Wahlström

**Affiliations:** 1 Department of Public Health Sciences, Division of International Health (IHCAR), Karolinska Institutet, Stockholm, Sweden; 2 Department of Health Affairs, Ministry of Health, Muscat, Oman; 3 Department of Family Medicine and Public Health, Sultan Qaboos University, College of Medicine and Health Sciences, Muscat, Oman; 4 Department of Molecular Medicine and Surgery, Endocrine and Diabetes Unit, Karolinska Institutet, Stockholm, Sweden

## Abstract

**Background:**

A good patient-physician interaction is particularly important in chronic diseases like diabetes. There are so far no published data regarding the interaction between the primary health-care providers and patients with type 2 diabetes in Oman, where diabetes is a major and growing health problem. This study aimed at exploring how health-care providers interact with patients with type 2 diabetes at primary health-care level in Muscat, Oman, focusing on the consultation environment, and some aspects of care and information.

**Methods:**

Direct observations of 90 consultations between 23 doctors and 13 diabetes nurses concerned with diabetes management during their consultations with type 2 diabetes patients in six primary health-care centres in the Muscat region, using checklists developed from the National Diabetes Guidelines. Consultations were assessed as optimal if more than 75% of observed aspects were fulfilled and sub-optimal if less than 50% were fulfilled.

**Results:**

Overall 52% of the doctors' consultations were not optimal. Some important aspects for a positive consultation environment were fulfilled in only about half of the doctors' consultations: ensuring privacy of consultation (49%), eye contact (49%), good attention (52%), encouraging asking questions (47%), and emphasizing on the patients' understanding of the provided information (52%). The doctors enquired about adverse effects of anti-diabetes drugs in less than 10% of consultations. The quality of the nurses' consultations was sub-optimal in about 75% of 85 consultations regarding aspects of consultation environment, care and information.

**Conclusion:**

The performance of the primary health-care doctors and diabetes nurses needs to be improved. The role of the diabetes nurses and the teamwork should be enhanced. We suggest a multidisciplinary team approach, training and education to the providers to upgrade their skills regarding communication and care. Barriers to compliance with the guidelines need to be further explored. Improving the work situation mainly for the diabetes nurses and further improvement in the organizational efficiency of diabetes services such as lowering the number of patients in diabetes clinic, are suggested.

## Background

Communication problems between patient and provider can cause difficulties in the effective delivery of health care [1]. A supportive consultation environment with a warm and caring physician, and a good patient-physician interaction is particularly important in chronic diseases like diabetes [2-4]. There is considerable evidence that many patients are not following an optimum management regime and that some reasons for this relate to the nature of the patient-doctor relationship [5]. The quality of care for patients with type 2 diabetes remains sub-optimal worldwide regardless of the country's level of development, efficacious treatments available, health-care system, or population characteristics and physician-patient communication has been judged to be generally inadequate [6,7]. The treatment of type 2 diabetes patients involves an effort on the part of the health-care team to impose new patterns of patient behaviour [8]. Nurses can make an important contribution for effective diabetes management in primary care [9].

Indicators of quality of care derived from international or national guidelines and with some kind of scoring system have been used to assess quality of communication and medical performance of health-care providers in several studies for different types of diseases [10-13].

Type 2 diabetes, with a prevalence of 11.6% in the year 2000, is a major and growing health problem in the Sultanate of Oman, due to the rapid socio-economic development including changing dietary patterns and decreased physical activities. During the last few years the Ministry of Health (MOH) has supported improvement in diabetes care, through financial support and by developing detailed guidelines for primary care facilities, where diabetes care is mainly delivered [14]. To date, no published data assessed the level of compliance with these guidelines at primary care level in Oman.

This study aimed at exploring and observing health-care providers' performance during their interactions with type 2 diabetes patients at primary health care-level in Muscat, the capital of Oman, focusing on the consultation environment, some aspects of provided care and health education.

## Methods

This is a cross-sectional, explorative study of on-going practice in diabetes care. It is part of a larger study, including patient interviews, focus group discussions with patients and providers, and the collection of metabolic parameters.

In the Muscat region, the capital of Oman, there are 18 primary health-care centres (PHCCs) which provide free health-care for the citizens. Six PHCCs were chosen to represent different geographical areas within the region with a population of around 632.000 out of the total population of 2.3 millions[15]. Five of the health centres were part of the Ministry of Health (MOH) institutions, while one health centre was part of the Sultan Qaboos University, Department of Family Medicine and Public Health, providing outpatient care to the university staff and their families. All the health centres had similar facilities for diabetes care.

The five PHCCs under MOH ran a diabetes clinic two days per week with 2–4 doctors working alternately, and 1–3 diabetes nurses, who met the patients prior to the doctor's consultations. The appointment lists included 17–25 patients per day. The health centre at the university ran a diabetes clinic once a week with six doctors alternately, and one diabetes nurse, and with 3–6 patients per day on the appointment lists. In four health centres, there was one health educator, and three health centres had one dietician, who received the patients on referral base.

All 23 doctors (14 men, 9 women) concerned with provision of care for the patients with diabetes in the six PHCCs and 13 women diabetes nurses participated after giving written consents. The doctors were of different nationalities (four Omani citizens, ten from other Arab countries, and nine from Asian and European countries), aged from 29 to 55 years, with general work experience not less than three years. The designation of the university doctors was family physician, while the MOH doctors are known as general practitioners or medical officers. Five doctors from the University PHCC had international diplomas regarding diabetes management, while 15 doctors from the other PHCCs had participated in short-term training. Ten of the diabetes nurses were Omani and three were of other Asian origin, aged from 25 to 40 years, with minimum three years of nursing experience, and with special training in diabetes care including health education. In three PHCCs, the diabetes nurses shared the office with the doctors.

In each PHCC, 15 consultations were observed, divided among the doctors who provided the diabetes care. Patients were selected according to the following inclusion criteria: (1) Type 2 diabetes, (2) Omani nationality, (3) Verbal consent.

Less than ten patients refused to participate, mainly due to time constraints.

The data were collected from January 2004 to August 2004 through direct observations (using checklists) of the doctors and diabetes nurses during their interactions with type 2 diabetes patients. The principal investigator (NA) performed all the observations in the six facilities. Each patient was followed during consultations with the nurse and doctor. Consultation time was recorded as well as use of translators. The health centres were visited on more than one occasion to enable observations of all concerned staff.

The checklists were developed by the research team, commented on by some heads of PHCCs, and thereafter modified. They included nine aspects of environment and atmosphere for doctors and nurses; eleven aspects of care provided by the doctors, including health education; and 19 aspects of care by the nurses including health education. The aspects are shown in tables 1, 2, and 3, respectively.

**Table 1:** Aspects of environment and atmosphere observed during consultations with the doctors and diabetes nurses

Consultation environment and atmosphere	Doctors' consultations n = 90 (%)	Nurses' consultations n = 85 (%)
Friendly welcoming*	67 (74 %)	41(48%)
Introductory chat	73 (81 %)	39 (46%)
Ensured privacy during consultation	44 (49%)	11 (13%)
Encouraged patients to ask questions	42 (47%)	9 (11%)
Attention all times	47 (52%)	20 (23%)
Gestures to continue†	72 (80%)	22 (26%)
Eye to eye contact	44 (49%)	19 (22%)
Emphasis on understanding and follow up	47 (52%)	14 (16%)
Friendly closing and fare well ‡	63 (70%)	16 (19%)

*Friendly welcoming was indicated if any of the following things occurred; a cheerful greeting with a smile, calling by names or shaking hands.

†This was considered positive if the provider was nodding his/her head while the patient was talking or if the doctor had vocal intonation to encourage the patient to continue.

‡Friendly closing and fare well were considered if the provider had some social talks with the patients at closing of the encounters or emphasised on what was discussed during the encounters; reassured the patient; asked the patient if anything else; said goodbye and thanked the patients.

**Table 2:** Aspects of care and information during 90 consultations with 23 primary care doctors

Aspects of care and information	Consultations n = 90 (%)
Asked about diet compliance	76 (84)
Inquired about physical activities	76 (84)
Emphasized on blood sugar control	75 (83)
Advised on healthy life (health education)	74 (82)
Asked about medicine compliance (81 patients)*	65 (80)
Asked about symptoms	71 (79)
Described how to use the medications (83 patients)*	65 (78)
Physical examination	64 (71)
Referred the patient to health educator or dietician	16 (18)
Asked about smoking and alcohol habits	8 (9)
Asked about adverse effects of medication (81 patients)*	7 (9)

*Nine patients were on diet control only; two of them were prescribed oral hypoglycaemic agents on the day of observation; seven patients were on insulin; 74 patients were on oral medication mainly sulphonylureas (76%)

**Table 3:** Aspects of care and information for 85 consultations with 13 diabetes nurses

Aspects of care	Consultations (%)
Measured blood sugar	82 (96)
Measured blood pressure	81 (95)
Measured height (in the first visit)	80 (94)
Measured weight	73 (86)
Reviewed the previous readings	38 (45)
Comments on the readings	36 (42)
Inquired about dietary habits	33 (39)
Inquired about physical activities	33 (39)
Inquired about medication compliance for 81 patients*	20 (25)
Calculated BMI (body mass index)	18 (21)

Aspects of information	

Education on foot care and self-hygiene	24 (28)
Provided printed educational materials	20 (23)
Emphasising importance of self-management†	17 (20)
Emphasising importance of diet control	16 (19)
Education on diabetes (symptoms, complications, management)	15 (18)
Emphasising importance of exercise	9 (11)
Emphasising importance of metabolic control	3 (3)
Education on hypoglycaemia	1 (1)
Emphasising importance of annual review for screening of complications	1 (1)

*81 type 2 diabetic patients who were on oral medication

† Self-management refers to changes/modifications in life style that help controlling the blood sugar like cooking process and preparing meals, amount of dates to be taken, exercise, stress management, home glucose monitoring, keeping record, monitor blood pressure, foot care and self-hygiene

All aspects of care and a few aspects regarding consultation environment were obtained from the clinical guidelines for diabetes management at primary health-care level, provided by the MOH in Oman. These guidelines describe the duties of the diabetes team and include a detailed list of recommendations for good quality care and health education that ought to be followed in each PHCC. The university PHCC had similar guidelines for diabetes management. Most of the aspects of good consultation environment were obtained from other similar studies and adjusted to the Omani context [7,16-23]. See footnote in table 1 for details.

The consultations were recorded using audiotapes for corroboration of some of the verbal communication aspects of the observations. The audiotapes were used by the Arabic-speaking members of the research team to confirm or challenge the ratings of the observer. The reliability of the observer's scorings was checked by comparison with two independent examiners, who made their ratings after listening to 33% (n = 30) of the audiotapes of the doctors' consultations (five at each PHCC) and 20% (n = 17) of the nurses' interactions. Some aspects that could not be observed through listening to the audiotapes were excluded. We found an acceptably high correlation between the external examiners expressed by a Spearman's rank correlation coefficient of 0.74–0.81 between the Observer and Examiner 1 and 2, respectively, for the doctors' consultations. There was a similar level of correlation for ratings of the nurses' interactions (0.78–0.87) between the Observer and Examiner 1 and 2, respectively.

The correlation coefficients between Examiner I and Examiner 2 were 0.78 and 0.81, respectively. Furthermore, in about half of the cases (16 and 9, respectively) the total scores of the observations of doctors and nurses by the Observer were lower than that of the other two examiners, while it was equal (n = 1 for both) or higher (n = 13 and 7) for 14 and 8 of the observations, respectively.

The data were entered into Excel. Each consultation with a doctor or diabetes nurse received a score for each aspect of the two areas of consultation environment and care, including health education. The score assessed the level of fulfilment or absence of the observed aspect. Each observed aspect was granted 1 point if completely fulfilled; 1.5 points if fulfilled sometimes and 2 points if not fulfilled. The total score per consultation was divided by the number of aspects in each consultation and mean values for scores per number of consultations were calculated for each individual doctor and diabetes nurse.

Optimum interaction was considered if the missed aspects were less than 25%, intermediate level of interaction for those who fulfilled 50–75% of the aspects and sub-optimal interaction if the health- care provider fulfilled less than 50% of the aspects.

At the PHCC level, the scores for all providers were summed up and divided by the total number of doctors and nurses in each PHCC. The range for optimum, intermediate and sub-optimal performance was determined using the same cut-off levels as for individual patients.

Association between performance and doctors' nationality, age, general work experience and special training in diabetes was analyzed with SPSS version 14, by using Fisher's Exact Test. Furthermore, association between performance and gender of doctors and patients was analyzed with Minitab program version 13, by using the Mann-Whitney U test on the median scoring of the doctors who received patients of the same sex versus those who received patients of different sex. Consultation time and patients educational levels in relation to doctor's performance were analyzed using the Kruskal-Wallis test.

Ethical clearance and approval was obtained from the Medical Research and Ethics Committee of Oman. Information sheets were sent to the health-care providers before the study started and their written consents were obtained. Verbal consents from the patients with type 2 diabetes were obtained after explanation of the study objectives and their anonymity was guaranteed.

## Results

Ninety patients consented to participation in the study. The age range was 35–75 years (mean = 53 years). Sixty percent of the patients were female, 51% were illiterate, 24% had primary education, and 24% had intermediate to university level education.

### Consultation environment and atmosphere

All 90 diabetic patients were consulted by the doctors, and 85 of them were seen by the trained diabetes nurses. There were variations in the ways in which the health-care providers interacted with the patients (Table 1).

A friendly welcoming of the patients was performed in 74% of all doctors' consultations and in 48% of the nurses' consultations. Privacy was not ensured in more than 50 % of the doctors' consultations and in more than 80 % of the nurses' consultations, as there were interruptions such as telephone calls, knocking on the doors and entering of other uninvited patients to doctors' and nurses' offices.

In about 50% of the consultations, the doctors paid full attention to the patients and emphasized verifying their understanding, while this was the case in less than 25% of the nurses' consultations.

Eye contact, while talking to the patients and encouraging patients to ask questions were performed in about 50% of doctors' consultations, but much less often in the nurses' consultations.

In nine consultations, four non-Arabic speaking doctors asked for interpretation from local health workers or colleagues. Nonetheless, this did not affect their performance as they interacted optimally with their diabetic patients. None of the diabetes nurses asked for interpretation. They either spoke the patient's language or used few Arabic words in a way that is familiar to the patients. However, all the diabetes nurses spent few minutes with the patients and the communication was short and quick in most of consultations.

The average length of the doctors' consultations was 10–15 minutes in 57 (61%) consultations, while it was less than 10 minutes in 24 (27%) and more than 15 minutes in 11 (12%) consultations.

### Aspects of care and information

#### The doctors

In more than 75% of the consultations, the doctors asked about symptoms, diet, medicine compliance, and physical activity, and provided health and dietary education, including education about diabetes complications and the importance of diet control and physical activity (table 2). Referrals to the health educators and dieticians were made in 18% of the consultations. In only 9% of the consultations, the doctors asked about adverse events of anti-diabetes medications like symptoms of hypoglycaemia, or inquired about smoking habits.

#### The diabetes nurses

Almost all the diabetes nurses measured weight, height, blood pressure and blood sugar in consistent manner, while the body mass index (BMI) was calculated in much fewer consultations (table 3).

Education on diabetes and the importance of self-management and foot care was provided in less than 18–28% of the consultations, whereas education on hypoglycaemia and importance of metabolic control was provided to 1–3 patients only.

### Summary scores of performance

#### The doctors

The overall scoring of the consultations showed that ten doctors were optimal in their interaction with the patients, both creating a positive consultation environment and providing optimal care and information, while nine doctors and four doctors performed at an intermediate or sub-optimal level, respectively. Forty-three (48%) of the patients had an optimal consultation environment and received optimal care and information, while the consultations were of an intermediate standard for 26 (29%) and of sub-optimal standard for 21 (23%).

Regarding all aspects, the doctors' performance were significantly better if they were over 40 (p = 0.003), and if they had more formal training in diabetes management (p = 0.004). However, there was no significant association between the doctors' performance and their nationality, their general work experience, or regarding the educational level of the patients. Furthermore, there were no significant differences in performance when male or female doctors interacted with a patient of the same or other sex.

Consultations of less than ten minutes' length had significantly lower scores than longer consultations (p < 0.001).

#### The diabetes nurses

The summary score for the diabetes nurses showed that three of them provided an intermediate consultation environment and care, while the other ten nurses interacted in a sub-optimal manner. Only one patient had an optimal consultation, 20 (24%) consultations were at an intermediate level and 64 (75%) were sub-optimal.

#### The health centres

The combined score for each PHCC showed that both doctors and diabetes nurses interacted optimally with type 2 diabetic patients in only one PHCC, which was the university health centre, while the interaction was sub-optimal in four of the other PHCCs and at an intermediate level in one.

## Discussion

Our findings show that overall slightly more than half of the interactions between doctors and type 2 diabetes patients were not optimal, in relation with the national guidelines, and that three out of four consultations with nurses were suboptimal. One reason could be that one out of four of the doctors' consultations and all consultations with nurses were shorter than ten minutes. It has been suggested that good communication inevitably takes more time that can be compressed only at the cost of the quality of care [24].

Other findings were that some important aspects for a positive consultation environment, like ensuring privacy during consultation, eye contact, good attention, encouraging the asking of questions, and emphasizing the patients' understanding of the provided information, were fulfilled in only about half of the doctors' consultations and even less often in the nurses' consultations.

A good consultation and patient-doctor communication demand uninterrupted privacy and undivided attention to the patient [25]. Privacy was interrupted during 10% of doctors' consultations in general practice in the UK [18], 30% in general practice in the Netherlands [17] and 33% in two health centres in Egypt [26]. Good eye contact and attentive listening have been observed as core processes of an effective patient/provider relationship [19].

Encouraging the patient to ask questions is not only a method of information seeking, but also a mechanism of patient participation (patient-centred care) and verifying understanding. It allows the patient's point of view to guide the conversation which has been shown to be positively associated with health outcomes [7,16,20]. It has been argued that training of health-care providers to be more patient-centred may improve communication in consultation and increase patients' satisfaction with their providers' manner [27]. We consider it a realistic possibility to adjust the model of patient-centred care to the Omani context by interventions at the level of the health-care providers to promote a patient-centred approach within clinical consultations, and by providing structured and continuous health education to the patients with diabetes to encourage them to participate in the medical dialogue.

The doctors asked about smoking and alcohol consumption habits in only one out of ten consultations. It was not clear if the patients had been questioned on this issue before, but risky habits should also be considered during regular consultations according to the guidelines [14]. Only one out of ten patients was asked about possible adverse effects of the anti-diabetes medication: these adverse effects might reduce patients' compliance. Better understanding of the drugs and dissemination of the information to doctors and patients has been shown to reduce the number of hypoglycaemia reactions caused by the sulphonylureas [28].

The majority of the doctors in this study were not acting as members of the diabetes team. They mostly provided the health education themselves and referred only a few patients to the health educators and dieticians, even if they were available in the health centres. These two categories of providers were not included in this study as they were not present in all health centres and they had a limited number of interactions with type 2 diabetes patients. However, teamwork for diabetes treatment and the nature of diabetes care result in a diffusion of responsibility for care from physicians to nurses, dieticians, and patients [29]. The poor collaboration between the doctors and other team members might indicate a weakness in the utilization of the available resources in the studied PHCCs. A multidisciplinary team approach is probably more effective and efficient [30].

We found no association between doctors' performance in relation to their gender and patients' gender regarding the overall interactions. These findings are supported by one large study on diabetes in primary care in US [31]. However, it has been found in other studies that female physicians in primary care generally communicate in a more patient-centred way than male physicians [32]. The findings related to the doctors' age and special training in diabetes management must be taken with great caution, as the number of doctors was limited.

The overall performance of the diabetes nurses in this study was sub-optimal. They had limited interactions with diabetes patients regarding the aspects of consultation environment, care and health education despite the description of their role and responsibilities in the national guidelines. In 7–9 out of ten consultations, the diabetes nurses did not provide education about good components of optimal diabetes care as recommended in the national guidelines [14]. The majority took the basic measurements and they did not comment on the weights of the patients although there is a high rate of overweight and obesity among Omani population [33]. The nurses to a great extent committed themselves to technical work and had quite limited interaction with the patients, contributing to the low scores. The reasons could partly be that in the studied health centres the diabetes nurses either shared the room with the doctors in three health centres or counselled the patients in the nurses' offices. Interruptions by other nurses and patients occurred in both situations, but were more common in the nurses' offices than in the doctors' offices.

However, ideally diabetes nurses can contribute to the teamwork by providing health education, and even assume some of the responsibilities of the physicians, if they are trained with detailed management protocols available [34], and if they have enough time [35]. It has been reviewed that the specialist diabetes nurse is in a unique position in that a relationship between patient and diabetes nurse could be maintained over a long period of time and can provide support for patients [36]. Providing special rooms for diabetes nurses in the PHCCs in Muscat may be helpful in ensuring privacy, contribute to increasing the autonomy and responsibilities of the diabetes nurses and allow interactive participation in teamwork through patients' counselling and provision of health education. In addition, continuous support to the nurses to actively take part in changes and development of services is recommended [37].

We cannot relate the overall low standard of care provided by the diabetes nurses in this study to language barriers as there were only a few non-Arabic speaking diabetes nurses. Similarly, in one study in the U.K, it was found that many diabetic patients received poor quality care, although this appeared to be related less to language difficulties than to professional attitudes and methods of working [38].

Four PHCCs scored sub-optimally, which can not be related to structural short-comings as all PHCCs had similar facilities for diabetes management. More probably it may be related to the total number of patients cared for [39], and the competence of the individual provider [40]. In one study, only doctors with a special interest in diabetes achieved significantly better outcomes than other doctors[41]. Lowering the patient: doctor ratio is important for improving the organizational efficiency of the diabetes service [42] and can be implemented in Oman.

A certain degree of observational bias is possible in this study as all observations were made by a single individual. Theoretically, two independent observers might have produced more reliable data. However, the performance of health-care providers may be affected by the fact that someone is making observations, positively or negatively, regardless of how sensitively observations are made [43]. Therefore, as these kinds of observations have not been done before in the health care services in Oman, we highly judged the importance of being as little intrusive as possible, favouring using only one observer. Furthermore, the consultation rooms are small, making it logistically difficult for more than one observer to be present. However, to make it possible to test for reliability of the observations and to allow other members of the research team to get some information about the actual interaction and to perform reliability testing, we decided to record all consultations on audiotapes. We did not consider video-taping as this was even more unfamiliar to the primary health care providers and would have influenced the consultations too much. Furthermore, pre-tests had been done before the start of the actual observations to ensure accurate and consistent performance of the observer.

There are some advantages of using only one observer. It means that all observations are made in a similar way and that the health care providers only need to meet one other person, who will then become less of a stranger and thereby probably influence the actual performance to a lesser extent.

We performed multiple observations with each doctor and nurse and found that after 1–2 observations, doctors and diabetes nurses' behaviour seemed not be affected or changed by having an observer in the consultation room. This finding is supported by the study of Parchman et al (2006), who also had one observer for all medical encounters in a diabetes clinic [44]. Furthermore, the audiotapes were used several times during the phase of data analysis by the Arabic speaking authors (including the observer) to confirm or revise the ratings. The additional reliability test by two independent examiners showed acceptably high levels of correlation and that the scorings by the observer were not systematically higher or lower than those of the independent examiners.

Other limitations could be that we developed a checklist that was designed to suit mainly the local settings regarding certain aspects and it might therefore lack validity to some extent. In addition, we aimed at assessing diabetes management from the perspectives of the health-care providers. However, Boon et al recommended validating the existing checklist designed by other researchers aimed at assessing the patient-doctor interaction [45]. Pendleton et al (1989) listed seven tasks in their consultation map that support a more patient-centred approach and ensure a positive consultation environment for both doctors and patients [46]. However, our approach was determined by the lack of similar studies in Oman and the need to get some basic information about the quality of provider services for better planning, potential need of interventions and maybe revision of the guidelines.

We consider that focusing on the health care providers' behaviours as an important factor in the process of communication and care at the preliminary stage of this on-going study will provide us with more information about an unknown situation in Oman. However, it has been argued that physicians appeared to make minimal efforts to foster patient involvement and autonomy that induce self-efficacy [2]. Furthermore, doctors' communication skills, hostility during interactions and training of health-care providers regarding interactions with patients and patient- centred care have been identified as crucial for effective health outcomes [2,20,27]. Moreover, focusing on the perspective of the patient, e.g., using Pendleton's mapping technique [46] could be the next step for more detailed studies of the consultation in the Omani context.

We know that a lot of barriers could affect the patient-doctor communication during consultations such as patients' literacy level [47,48]. However, patients' barriers or non-adherence can be changed by improving education, perception, motivation and self-management [49].

Due to limited size of the sample of the study population, the study is merely explorative. However, there is no standardized way of calculating sample size for explorative investigations and other studies have used similar numbers of observations per facility [10,50,51]. Furthermore, this study can not be generalised to the whole of Oman, but it provides some indications of how the situation might look like in other health centres with similar facilities or in the remote PHCCs. Moreover, our findings also concur with other studies worldwide [6,7].

Our findings show that compliance with the national guidelines on diabetes care was partly sub-optimal. It is known from previous research that dissemination of guidelines alone is not enough to change provider behaviour permanently [52]. However, lack of compliance with guidelines may indicate short-comings in physician knowledge, implementation problems, lack of belief in guidelines, or problems in patient compliance [53]. We suggest that attention and further exploration should be directed to all these areas before revision of the guidelines. More interactive methods such as audit and feedback can be effective in improving professional practice [54,55], and would be useful also in Oman.

## Conclusion

The performance of the doctors and diabetes nurses needs to be improved. The role of the diabetes nurses and the teamwork should be enhanced. We suggest a multidisciplinary team approach and further training and education of the health-care providers to upgrade their skills regarding communication and care in accordance with what has already been developed in the national guidelines. Barriers to compliance with the guidelines need to be further explored. Improving the work situation mainly for the diabetes nurses and further improvement in the organizational efficiency of diabetes services such as lowering the number of patients in diabetes clinics, are suggested.

The results of this study can provide a basis for further studies concerning diabetes care at the primary care level in Oman and countries with similar health systems. The results can also be used as a material for educational interventions.

## Abbreviations

MoH – Ministry of Health, PHCC – Primary Health Care Centre

## Competing interests

The author(s) declare that they have no competing interests.

## Authors' contributions

NA conceived the study, participated in its design, conducted the observations, analysed the data, drafted and finalized the manuscript. MAA participated in discussing the concept of the study and in critical review of the manuscript; CGÖ and RW participated in the design of the study, data analysis, discussing, rewriting draft versions and critically revised the manuscript. AV participated in discussing the concept of the study and performed the statistical analysis. All authors read and approved the final manuscript.

## Pre-publication history

The pre-publication history for this paper can be accessed here:


